# Attention, working memory, and phenomenal experience of WM content: memory levels determined by different types of top-down modulation

**DOI:** 10.3389/fpsyg.2015.01603

**Published:** 2015-10-19

**Authors:** Jane Jacob, Christianne Jacobs, Juha Silvanto

**Affiliations:** Department of Psychology, Faculty of Science and Technology, University of Westminster, London, UK

**Keywords:** working memory, consciousness, top-down attention, phenomenal experience, memory state

## Abstract

What is the role of top-down attentional modulation in consciously accessing working memory (WM) content? In influential WM models, information can exist in different states, determined by allocation of attention; placing the original memory representation in the center of focused attention gives rise to conscious access. Here we discuss various lines of evidence indicating that such attentional modulation is not sufficient for memory content to be phenomenally experienced. We propose that, in addition to attentional modulation of the memory representation, another type of top-down modulation is required: suppression of all incoming visual information, via inhibition of early visual cortex. In this view, there are three distinct memory levels, as a function of the top-down control associated with them: (1) Nonattended, nonconscious associated with no attentional modulation; (2) attended, phenomenally nonconscious memory, associated with attentional enhancement of the actual memory trace; (3) attended, phenomenally conscious memory content, associated with enhancement of the memory trace *and* top-down suppression of all incoming visual input.

## Introduction

In influential models of working memory (WM), information can exist in different states which are determined by the allocation of attention. The influential model of Cowan (1988) conceptualizes WM as activated long-term memory, able to retain a number of activated representations in parallel, including re-enacted representations from long-term memory. In a development of this model, Oberauer (2002) introduced a store of reactivated long-term memory representations, a capacity-limited short-term store (or zone of direct access), and a process of focused attention with a maximal storage capacity of a single item only (Oberauer, 2002, 2009). However, only representations in the focus of attention (FOA) can be directly accessed for goal-directed processing (Cowan, 1988). At the neural level, these attentional effects appear to be manifested as top-down modulations of neural representations in the sensory cortex which are involved in the maintenance of a given stimulus. For example, in a study on visual WM, Lewis-Peacock and Postle (2012) found increased decoding accuracies for attended versus unattended memory items in the visual cortex; furthermore, the unattended WM content could not be decoded from the BOLD signal, indicating that the attended and nonattended memory items are in different representational states in the visual cortex. The neural signature of the FOA appears to be fronto-parietal modulation of the neural representations in sensory regions engaged in memory maintenance (Feredoes et al., 2011; Riggall and Postle, 2012; Emrich et al., 2013).

**FIGURE 1:**
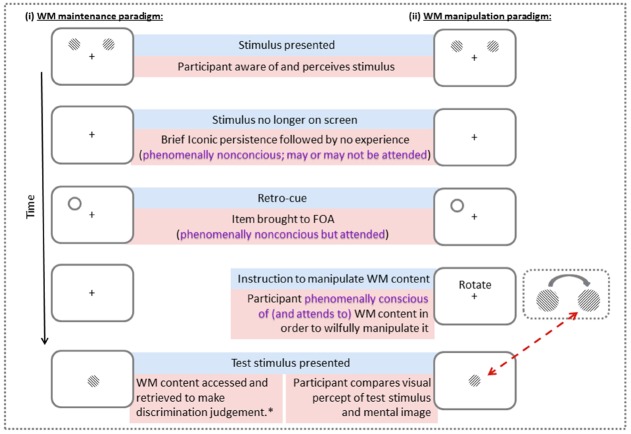
**Depiction of the different possible stages of phenomenal experience in WM tasks for (i) WM maintenance paradigm and (ii) WM manipulation paradigm.** Note that when the task requires interaction with the WM content, the item becomes phenomenally conscious. On the other hand, in the maintenance task, the participant is phenomenally conscious of the WM content when making a discrimination judgment at the end. *Some participants may use imagery to perform the task, thus it may involve phenomenally conscious WM content. In other words, when they are not instructed to use imagery, we cannot be certain that they do not engage in it.

While the state-based models have been effective in explaining the role of attention in accessing WM content, they have not addressed the issue of how this information is brought to *phenomenal* experience (see Jacobs and Silvanto, 2015, for review). Yet a key aspect of WM is that its content can be experienced in the form of an image containing qualia, which can be consciously scrutinized and manipulated; for example, modifying WM content on the basis of explicit instructions requires such a conscious process (see Figure 1). We stress that we do not refer to the conscious experience of the memory cue, but rather the process of bringing information from outside of FOA to phenomenal experience in order to be inspected and manipulated.

Here we propose that attentional modulation of the actual memory trace is not by itself sufficient for the memory content to be phenomenally experienced. We identify three distinct memory levels, classified by the top-down control associated with them: (1) nonattended nonconscious memory, associated with no attentional modulation; (2) attended nonconscious memory, associated with attentional modulation of the actual memory representation; (3) attended, phenomenally conscious memory content, which (in addition to attentional enhancement of the actual memory trace) involves top-down suppression of all incoming visual input. Our view differs from prior models in that there are distinct nonconscious and conscious attended memory states.

## Evidence Indicating Dissociation between Attention and Phenomenal Experience of Memory Content

### Nonconscious Items Can be Attended

A key finding pointing toward a dissociation between attention and consciousness in the context of WM is that, in the absence of awareness, items can be attended, encoded into and maintained in WM. In a study by Soto et al. (2011), participants were able to encode and maintain masked gratings in memory and use these to perform at above-chance level in a delayed forced-choice discrimination task, despite reporting being unaware of cue presence. Above-chance discrimination performance was found even when distracters were presented during the maintenance period; this resistance to distracter influence indicates that attention was allocated to memory content (i.e., it was in the focus of attention) throughout the delay period.

One might raise the question of whether it can be assumed that items in WM are attended simply because there is only one item to keep track of. What speaks in support of the view that the items held nonconsciously in WM are in fact attended is that the above-chance discrimination performance found even when distracters were presented during the maintenance period. This resistance to distracter influence indicates that attention was allocated to memory content (i.e., it was in the FOA) throughout the delay period. Feredoes et al. (2011) demonstrated that the survival of a WM trace in the presence of distracters is accomplished by top-down facilitation of visual cortical regions maintaining the WM content. It is therefore parsimonious to argue that nonconscious WM content benefits from this kind of top-down modulation. Studies explicitly manipulating attention, as used in the study of visual perception (e.g., Kanai et al., 2006) are required to demonstrate this issue conclusively.

There are various other examples of nonconscious WM effects. Using continuous flash suppression, Pan et al. (2014) showed that participants were able to make discrimination judgments based on WM representations of unseen stimuli during interocular suppression. Bergström and Eriksson (2014) used a rapid serial visual sequence (from the attentional blink phenomenon) to render letters as nonconscious, and demonstrated above chance performance in WM maintenance of the letters of which participants reported as being unaware. These behavioral findings are further supported by fMRI studies which have found BOLD signal changes in right mid-lateral PFC, OFC, and cerebellum associated with maintaining nonconscious WM representations (Bergström and Eriksson, 2014). Dutta et al. (2014) report activation in the dorsolateral prefrontal cortex (DLPFC), anterior PFC (aPFC) and posterior parietal regions when maintaining nonconscious information in visual WM (Dutta et al., 2014). Thus prefrontal regions normally associated with attentional top-down control were engaged in maintenance of nonconscious items (see Soto and Silvanto, 2014, for review). Furthermore, there is also evidence for dissociation between attended and conscious states, where subliminal shapes in visual WM guide attention and facilitate WM performance (Astle et al., 2010). Based on these studies, it is evident that WM can maintain nonconscious representations, i.e., information in the FOA in WM is not necessarily conscious.

The major implication of these studies is that attention can be allocated to WM without conscious experience of memory content, pointing toward a dissociation between conscious access and attentive memory states. While we do not challenge the view that attention is required for memory contents to be phenomenally experienced, we suggest that additional processes are required for this to occur.

### Qualitative Differences Between Conscious vs. Nonconscious Memory Content

A further reason to suspect a dissociation between attended and conscious memory states/representations is that once memory content is brought to phenomenal awareness in the form of a conscious mental image, it interacts very differently with external input than memory content of which participants are not phenomenally aware, even though both are likely to be attended. Conscious experience of memory content in the form of mental imagery has been shown to interfere with the encoding of concurrently presented visual information. When participants are asked to form a conscious mental image of a recently presented item (thus phenomenal experience reflecting WM content), detection threshold of a concurrently present external visual target is elevated (Craver-Lemley and Reeves, 1987). Such effects are found regardless of the similarity between the mental image and visual input and are thought to be functionally important because it protects the mental image from being weakened by external information (Craver-Lemley and Reeves, 1992). This inhibition occurs also in the opposite direction, with external input impairing conscious experience of memory content, even when the two are identical (Bona et al., 2013). In short, phenomenally experienced WM content and externally presented visual information are in an inhibitory relationship, regardless of any congruency or similarity between the items.

The interaction with external input is fundamentally different for memory items which are presumably attended but not required to be phenomenally experienced (i.e., when there is no need to create a phenomenal mental image). Specifically, encoding of external stimuli matching WM content is enhanced, whereas those incongruent with WM content are suppressed (e.g., Downing, 2000; Soto et al., 2005; Pan and Soto, 2010; Carlisle and Woodman, 2011; Gayet et al., 2013). Moreover, when incoming visual information matches the content of WM, the former will reach visual awareness more effectively; in contrast, non-matching visual input is suppressed (Pan et al., 2014).

In summary, conscious experience of memory content in the form of visual imagery suppresses further processing of any visual input, whereas in the absence of imagery, WM can enhance the encoding of congruent visual input. It is parsimonious to assume that the memory item was attended in these studies, as there was always only one item to be held in memory and the task instruction stressed the importance of successful maintenance, and the memory items survive distracter presence. Yet the interaction between external input and internal attentional processing varied depending on whether the task required phenomenal experience of the WM item (i.e., the creation of a conscious mental image; see Figure 1). This suggests that there are different types of attended states (phenomenally conscious and nonconscious), with different functional characteristics.

It might be argued that these differential effects on concurrent sensory processing can be explained simply in terms of “more” vs. “less” attention available to WM items. In this view, engaging in imagery may require additional attentional resources, which could otherwise be allocated to the encoding of sensory input. This view fails to explain the findings discussed above which point toward a *qualitative* shift when imagery is engaged. Specifically, when the WM item is not imagined, there is a congruency effect such that input matching WM content is facilitated and incongruent items are suppressed. Imagery does not modulate this congruency effect in a quantitative manner but rather abolishes it altogether, with all incoming information being suppressed.

## Three Levels of Memory: Nonattended Nonconscious, Attended Nonconscious, and Attended Conscious

As discussed above, attention can be allocated toward nonconscious items, demonstrating that attention by itself is not sufficient for WM content to be consciously experienced (as has previously been discussed in relation to conscious experience of external input; cf. Lamme, 2003). Furthermore, phenomenally experienced memory content can have different functional properties compared to attended memory content that is not conscious; as discussed above, conscious experience of memory content in the form of visual imagery suppresses all visual input, whereas in the absence of imagery, WM acts as “gatekeeper” of visual input. Thus, there appears to be more to conscious experience of WM content than an “attended state” (e.g., Cowan, 1988, 1998; Oberauer, 2002; LaRocque et al., 2013).

**FIGURE 2:**
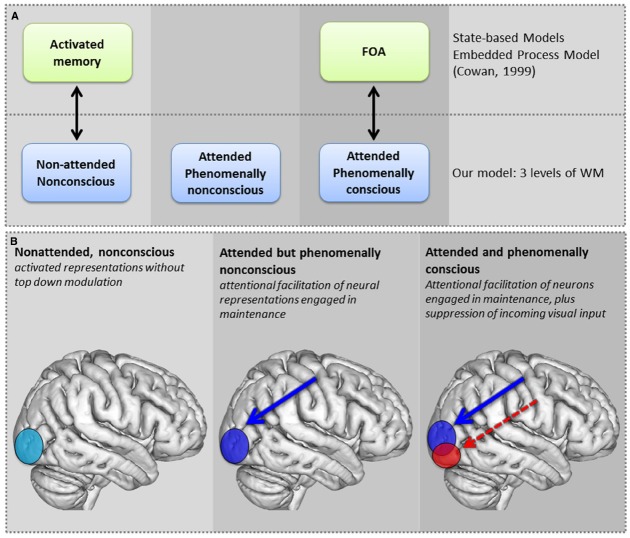
**(A)** Traditional and proposed models of WM: The top row illustrates the bipartite distinction of state-based models between activated representations of LTM, which are nonconscious, and attended representations in the FOA in WM, which are consciously processed. The bottom row illustrates the proposed three levels of WM which range from nonconscious representations that are not attended, to those that are attended, and finally, attended and conscious WM representations. Note that the state-based model does not account for the intermediate attended but nonconscious level of WM. **(B)** Three levels of WM processing are proposed—(1) Nonattended, nonconscious: activated representations without top down modulation, (2) Attended but nonconscious: attentional facilitation of neural representation engaged in maintenance, (3) Attended and conscious: attentional facilitation of neural representation engaged in maintenance plus suppression of early visual cortex of incoming input. This suppression could happen in early visual cortex or even at lower levels, as attention has been shown to modulate LGN as well.

To account for these phenomena, we propose that there can be both conscious and nonconscious attended memories, determined by the type of top-down attentional modulation that is being allocated (as discussed in the next section). In this view, there are three levels of memory: *nonattended nonconscious memory*, *attended nonconscious memory*, and *attended*, *phenomenally conscious memory* (see Figure 2). The first level of memory refers to traditional nonconscious processing of information that is not attended, and would be consistent with the activated portion of the long-term memory in Cowan’s and Oberauer’s models. The second level of memory involves nonconscious processing of information when attention is directed to it. In the context of visual perception, there is plenty of evidence of such effects (see Lamme, 2003, for review). In WM, items can be attended and nonconscious during the maintenance period when there is no need for conscious examination of memory content. They can also be in this state when the item was subliminal to begin with (cf. Soto et al., 2011). The third level of memory, which is conscious and attended, is where participants need to phenomenally experience memory representations (in other words, to create a conscious mental image) in order to inspect and to manipulate them.

The present proposal reflects the view that attention and awareness are distinct concepts which do not need to go hand-in-hand (Lamme, 2003; Koch and Tsuchiya, 2007; Van Boxtel et al., 2010). While conscious experience of WM content is likely to require attention, it does not appear to be sufficient for it. This notion is in accordance with the global workspace theory (GWT), which states that information held in the global workspace is conscious, and selective attention determines what information reaches consciousness (Baars, 2005). Dehaene et al. (2006) deviate from the dichotomy of conscious and nonconscious processing to a tripartite distinction, stating that information can be subliminal, preconscious or conscious. This view divides the traditional notion of unconscious processing into subliminal and preconscious states based on the absence of bottom-up or top-down attention, respectively. Information is subliminally processed when there is limited bottom-up processing due to attenuated stimulus strength, potentially interacting with top-down attention. This state is an example of attention not predicating consciousness. On the other hand, with stronger stimulus strength, but limited or no top-down attention, information undergoes preconscious processing, a state in which information has the potential for conscious access, but is not yet conscious. Conscious processing, according to this model, involves top-down attention and bottom-up activation past the sensory threshold. Similarly, state-based WM models assign conscious properties to the FOA and classify activated LTM representations as being nonconscious (Cowan, 1999; Oberauer, 2002).

While the GWT is not a model of WM but rather a theory of how visual input reaches awareness, we nevertheless discuss it here due to its importance in providing a theoretical division between attention and consciousness; there is value in attempting to translate its components to the context of WM. Our proposal is not a direct transfer of this model to WM, in that in the GWT, subliminal but attended information processing is short-lived, whereas we propose that in WM, nonconscious attended representations may be maintained for longer durations and survive distracter presence as seen in previous studies (Soto et al., 2011; Pan et al., 2014; Bergström and Eriksson, 2014; Dutta et al., 2014). Thus, the proposed “attended but nonconscious” level does not have an exact match in the GWT.

## Memory Levels Determined by the Type(s) of Top-Down Attentional Modulation

In various models of WM, attentional modulation of neural representations encoding the memory content (in other words, placing the memory item into FOA) enables conscious experience of that content. We propose that for WM content to reach phenomenal awareness in the conscious mental image, additionally another type of top-down modulation is required: the suppression of incoming visual information by inhibition of earliest visual processing (see Figure 2).

The need for such suppression arises from the fact that during conscious perception, it is critical to ascertain the source of conscious experience, in order to avoid confusion between internal and external sources of information. Such confusion can occur if internally generated and externally induced percepts are not kept apart, as observed in the Perky effect (i.e., the phenomenon where external input is confused to be part of mental imagery; Perky, 1910). Therefore, when WM content needs to be brought to phenomenal awareness, it is important that external information is not concurrently consciously experienced. The suppression of any incoming feedforward input prevents confusion between internally generated and externally induced perception, by preventing the latter from reaching conscious experience (see last panel in Figure 2B).

Why is this suppression needed? Under normal circumstances, a strong external stimulus is likely to win the competition for consciousness, as it induces stronger neural activity than visual imagery of the very same item (e.g., Kreiman et al., 2000). For this reason, when WM content needs to be brought to phenomenal awareness, the competition for consciousness needs to be biased in its favor. Suppression of incoming external input is thus a strong contributing factor in conscious experience of WM content.

As discussed in “Evidence Indicating Dissociation Between Attention and Phenomenal Experience of Memory Content,” engaging in imagery suppresses the encoding of incoming visual information (e.g., Craver-Lemley and Reeves, 1987). There is also neural evidence consistent with this view. In an fMRI study requiring participants to engage in imagery of visual motion, Kaas et al. (2010) found that activity in the early visual cortex was reduced during imagery, whereas it was enhanced in the motion-selective extrastriate region V5/MT (Kaas et al., 2010). It is interesting to note that the same early visual areas do show an increase in BOLD signal when motion information is held in WM without the need to phenomenally experience that memory content (Goebel et al., 1998; Slotnick et al., 2005). This is consistent with the view that the requirement to phenomenally experience an internally generated image is associated with suppression of early visual areas. Given that this type of suppression is only observed when consciously experiencing WM content, it is not to be mistaken simply as distractor inhibition mechanism, since it is not observed during conventional WM tasks that do not require imagery.

It needs to be noted that, at the behavioral level, distracter suppression can happen for items which we would argue to be attended but nonconscious; indeed, a hallmark of WM is that its content survives the presence of distracters. However, the key issue here is the neural implementation. As discussed above, the survival of a WM trace in the presence of distracters is implemented by strengthening neural activity in regions encoding the memory target, rather than by suppressing neural activity associated with distracters (Feredoes et al., 2011). We propose that indiscriminatory *neural* suppression of all incoming visual input at earliest levels of visual processing is a type of top-down modulation tightly linked to phenomenal experience of WM content.

Thus, the gist of our proposal is the following: behavioral suppression of distracters in conventional WM paradigms is accomplished by neural facilitation of the memory trace. In imagery, an additional mechanism is involved: neural suppression of all incoming input at the earliest levels of visual processing. As shown in Figure 2, this gives rise to the following levels of memory (whether these exist as separate states or as multiple representations requires further study):

(1) ***Nonattended*:** associated with no attentional modulation
(2) ***Attended nonconscious memory content*:** at the neural level, associated with top-down attentional modulation of the actual memory representation
(3) ***Attended, phenomenally conscious memory content*:** at the neural level, associated with attentional enhancement of the actual memory representation AND top-down suppression of incoming visual input

## Concluding Remarks

The aim of this article has been to clarify the relationship between top-down attentional control in phenomenal experience of WM content. Recently, we have argued that phenomenally experiencing WM involves the creation of a second, distinct, representation of the memory content (Jacobs and Silvanto, 2015). This raises the question of how the two top-down processes described above relate to the creation of this “conscious copy.” Possibly, top-down facilitation of the original memory trace is the process through which the generation of the second representation is achieved, while additional suppression of external visual input is required for this copy to win the competition for consciousness. Another open empirical question is the neural basis of the different processes and representations involved in phenomenal awareness of WM content. This could be addressed by a multi-voxel pattern analysis (MVPA) study which manipulates the level of phenomenal conscious access during a delay period in which distracters are presented. For example, participants could be shown a display of items, and later cued to consciously access one of the memory items and hold it in conscious phenomenal experience throughout the delay. In another similar condition, participants would be cued to maintain one of the memory items in WM, but not be required to phenomenally experience the item during maintenance (similar to most retro-cue WM discrimination tasks). During the delay, distracters would be presented. We would predict that, in both conditions, there would be stronger neural representation in regions maintaining the WM content. However, only in the “phenomenal awareness” condition, there would be a suppression of early visual regions as well as any extrastriate region selective for the distracter.

### Conflict of Interest Statement

The authors declare that the research was conducted in the absence of any commercial or financial relationships that could be construed as a potential conflict of interest.
